# Case Report: Differential response to IL-13 inhibition: lebrikizumab following primary tralokinumab failure in atopic dermatitis

**DOI:** 10.3389/fmed.2026.1953586

**Published:** 2026-09-23

**Authors:** Shukry Abdi, Clare Bailey, Andreea Cifrea

**Affiliations:** Department of Dermatology, Chelsea and Westminster Hospital NHS Foundation Trust, London, United Kingdom

**Keywords:** atopic dermatitis, biologic switching, case report, IL-13 inhibition, lebrikizumab, tralokinumab

## Abstract

**Background:**

Atopic dermatitis is a chronic inflammatory skin disorder driven largely by type 2 immune pathways, with interleukin-13 (IL-13) playing a central role in disease pathogenesis. Biologics targeting IL-13, including tralokinumab and lebrikizumab, have demonstrated efficacy in clinical trials; however, the therapeutic value of intra-class switching following primary treatment failure remains poorly defined, and existing real-world reports rarely distinguish primary from secondary failure. This gap is particularly relevant for patients with limited systemic treatment options, in whom treatment sequencing decisions carry greater clinical weight.

**Case presentation:**

We report a 29-year-old man with lifelong moderate atopic dermatitis and limited systemic treatment options due to contraindications and prior treatment inefficacy. He demonstrated confirmed primary failure to tralokinumab, with persistent disease activity, despite good adherence. Prior to switching to lebrikizumab, EASI was 9, POEM 16, and DLQI 8, with the patient achieving marked improvement in disease severity within four months (EASI 0, POEM 5, DLQI 7), alongside early improvement in pruritus. At six months, disease control remained substantially improved (EASI 1.4), with good tolerability and no requirement for rescue systemic therapy.

**Discussion:**

Despite targeting the same cytokine, tralokinumab and lebrikizumab exhibit distinct receptor-binding properties, with lebrikizumab preserving IL-13 interaction with the IL-13Ra2 decoy receptor, potentially enhancing cytokine clearance and modulating downstream signalling. This case demonstrates a clinically meaningful improvement in disease severity following primary failure to tralokinumab, supporting the concept of functional heterogeneity within IL-13 inhibition and suggesting that primary failure does not imply class-wide inefficacy.

**Conclusion:**

This case provides evidence that primary failure to tralokinumab may not preclude response to lebrikizumab, possibly supporting intra-class heterogeneity among IL-13 inhibitors. Sequential IL-13 inhibition may represent a rational therapeutic option in patients with limited treatment options. Further studies are needed to identify predictors of response and optimise biologic sequencing in atopic dermatitis.

## Introduction

Atopic dermatitis (AD), commonly referred to as eczema, is a chronic relapsing inflammatory skin disease characterised by pruritus, xerosis, and eczematous lesions (1). The pathophysiology of AD remains complex and multifactorial, involving epidermal barrier dysfunction and immune dysregulation, predominantly driven by type 2 inflammation pathways (2). Interleukin-13 (IL-13) has been identified as a key cytokine in the disease pathogenesis (3, 4), contributing to inflammation, barrier impairment, and pruritus. Advances in understanding these pathways have led to the development of targeted therapies, including Janus kinase (JAK) inhibitors, IL-4 inhibitors, and IL-13 inhibitors.

Real-world reports of switching from tralokinumab to lebrikizumab are emerging. However, existing reports do not consistently distinguish between primary and secondary treatment failure (5), limiting interpretation of treatment sequencing. We present a case of moderate AD with restricted systemic treatment options demonstrating marked clinical improvement with lebrikizumab following confirmed primary tralokinumab failure.

## Case presentation

A 29-year-old man with lifelong AD presented with persistent moderate disease. He was a social smoker and had a history of increased alcohol intake, and a body mass index >25 kg/m^2^.

Previous treatment for AD included unsuccessful phototherapy and conventional systemic therapies. Methotrexate was contraindicated due to elevated liver function tests (LFTs), and ciclosporin was contraindicated due to high blood pressure. He was subsequently commenced on upadacitinib; however, this was discontinued following a rise in LFTs. Subsequent gastroenterology assessment identified hepatic steatosis, likely related to alcohol consumption. As LFTs remain elevated, multidisciplinary discussion concluded that further JAK inhibitor therapy was not pursued because of the class-wide requirement for hepatic monitoring and risk of further transaminitis. Given the patient's facial atopic dermatitis, dupilumab was not selected in view of reported association with head-and-neck dermatitis and facial erythema (6, 7). Therefore, treatment decisions were guided by cumulative disease burden and limited systemic treatment options.

He was commenced on tralokinumab in November 2023 with a loading dose of 600 mg subcutaneously, followed by 300 mg injections every two weeks until stable. Prior to commencing tralokinumab, baseline disease severity was Eczema Area and Severity Index (EASI) of 14.4, Patient-Oriented Eczema Measure (POEM) of 25 and Dermatology Life Quality Index (DLQI) of 21. Topical corticosteroid and tacrolimus were used intermittently, with use occurring over a period of approximately two weeks per month when required, with additional use during significant flares. Despite good adherence, the patient reported little perceived improvement during the initial treatment period, and disease severity fluctuated without a sustained response, with persistent facial eczema and recurrent flares throughout treatment. Serial EASI scores remained variable during treatment, fluctuating between 8.1 and 16, POEM between 16 and 25, and DLQI between 8 and 21, without achieving a ≥ 50% reduction in EASI from baseline over the treatment period, consistent with primary treatment failure. Given the limited systemic treatment options, a decision was made in December 2024 to switch to lebrikizumab, however, treatment initiation was delayed until June 2025 due to supply issues, during which time tralokinumab was continued with no change in clinical status.

Prior to commencing lebrikizumab, the patient presented with excoriated, lichenified eczema predominantly affecting the upper body (Figures 1, 2). Baseline disease severity was moderate, with an EASI score of 9, a POEM score of 16, and a DLQI score of 8. Pruritus score was not formally quantified at this time but was documented as significant.

**Figure 1:**
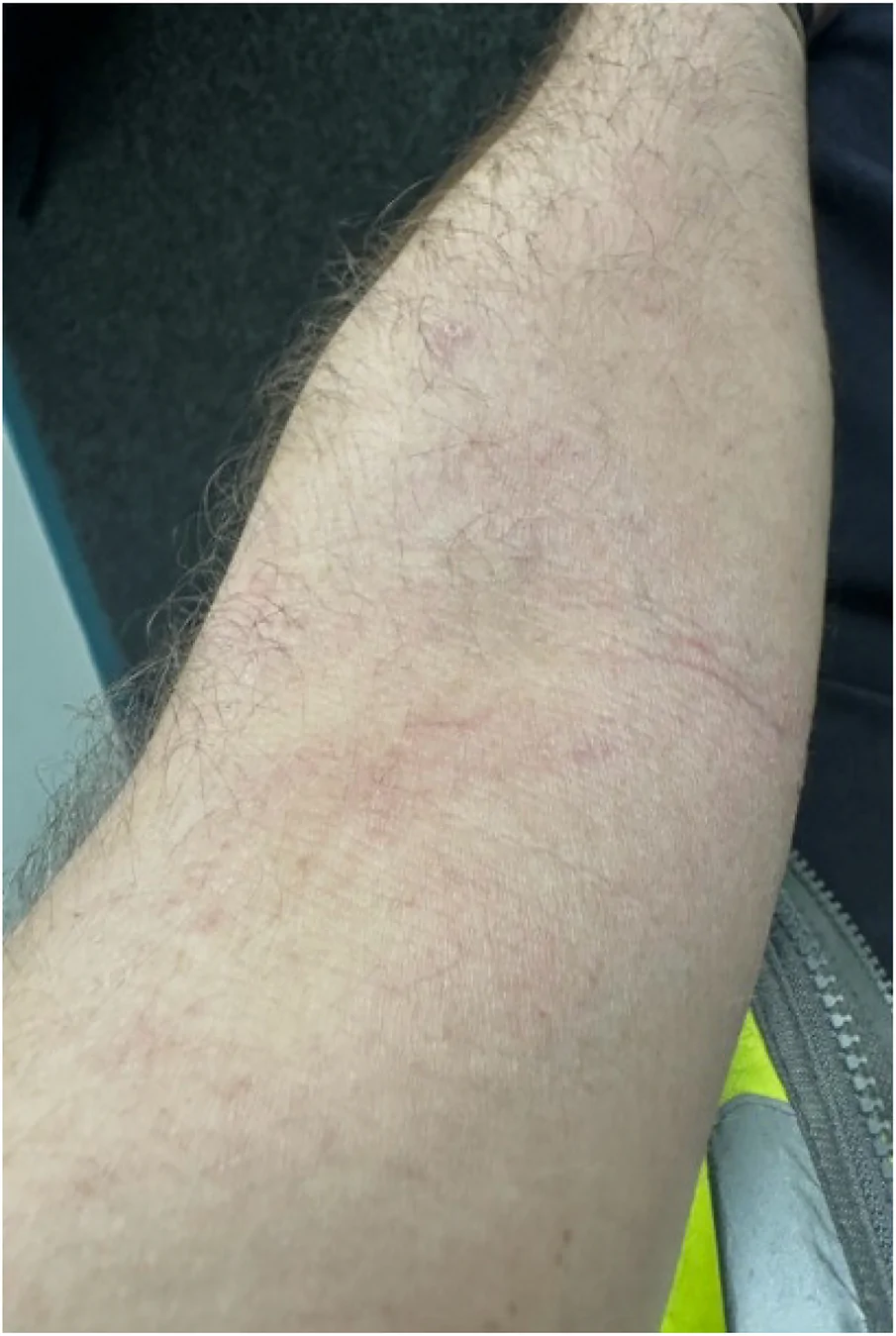
Upper limb involvement demonstrating excoriated, lichenified eczema, prior to initiation of lebrikizumab.

**Figure 2:**
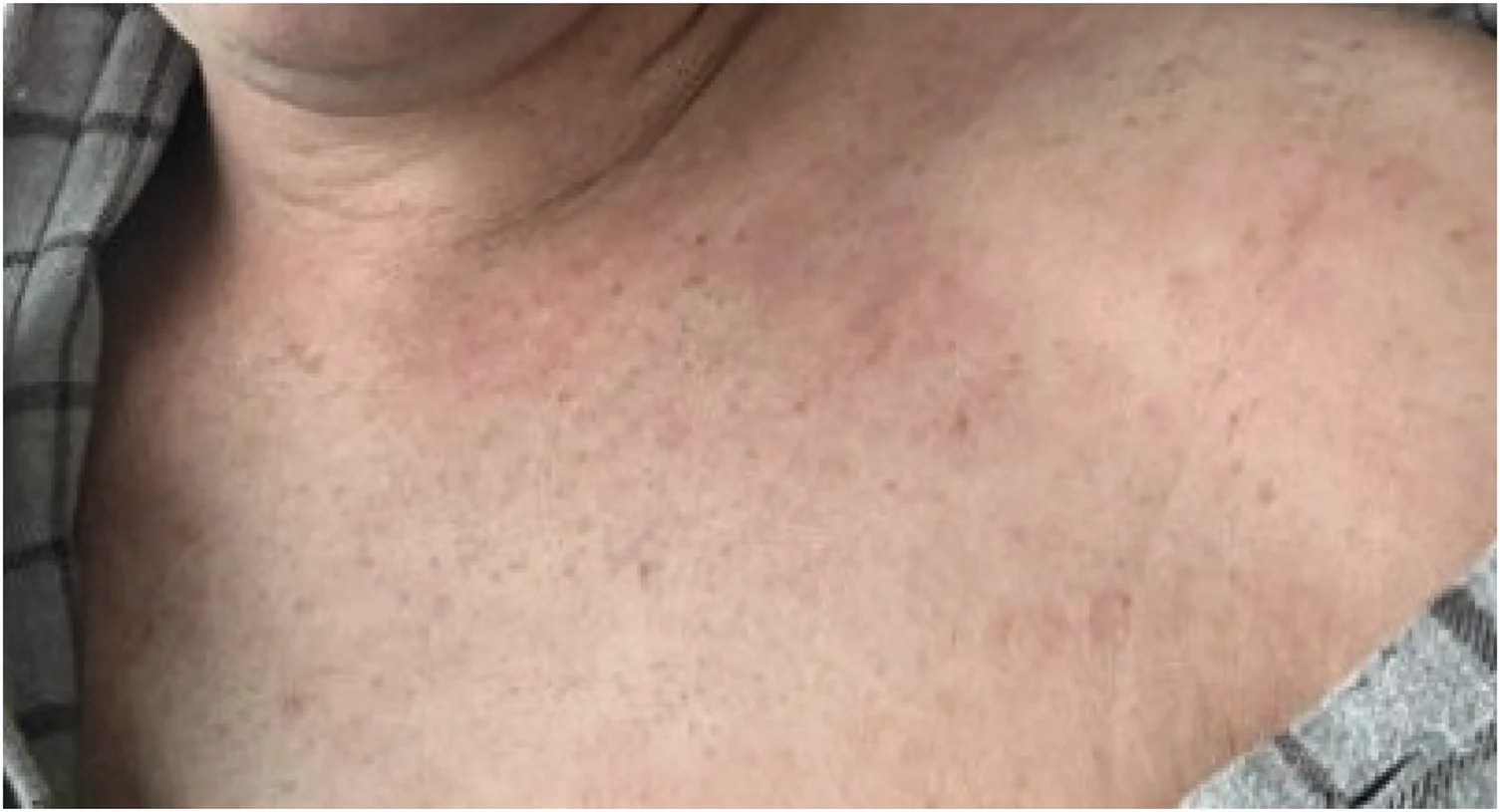
Upper body involvement demonstrating mild erythema with scattered eczematous lesions prior to lebrikizumab initiation.

Lebrikizumab was initiated with a loading dose of 500 mg, followed by 250 mg every two weeks until week 24, followed by 250 mg every four weeks thereafter. No washout period was implemented between the two biologic treatments, reflecting real-world practice and minimising the risk of disease flare. Topical corticosteroids and tacrolimus continued to be used intermittently at a similar frequency to that used during tralokinumab treatment, with no significant flares occurring during this period.

Four months following the switch, the patient demonstrated marked clinical improvement with an EASI score of 0, a POEM score of 5, and a DLQI score of 7. The patient also reported significant improvement in pruritus, with a pruritus Numeric Rating Scale (NRS) score of 3.

At six-month follow-up, overall disease control remained substantially improved compared to baseline, with a more recent EASI score of 1.4, POEM score of 9, DLQI score of 5, and a pruritus NRS score of 6. Minimal residual eczema remained, predominantly affecting the face and left antecubital fossa (Figures 3, 4). Additionally, the patient tolerated lebrikizumab without treatment-related adverse side effects.

**Figure 3:**
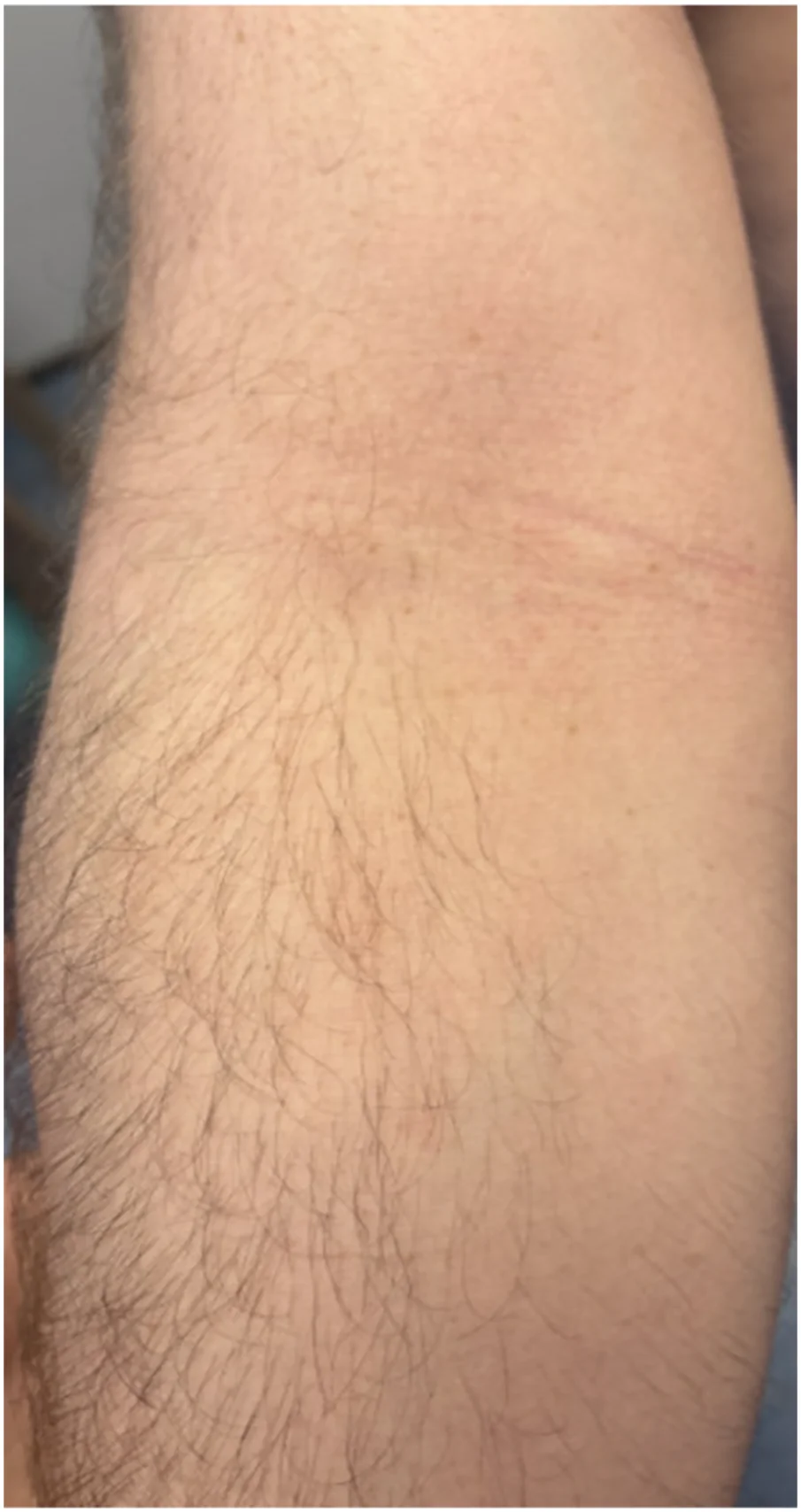
Upper limb involvement at 6 months follow-up demonstrating near-complete resolution of eczema, with no residual excoriation or lichenification.

**Figure 4:**
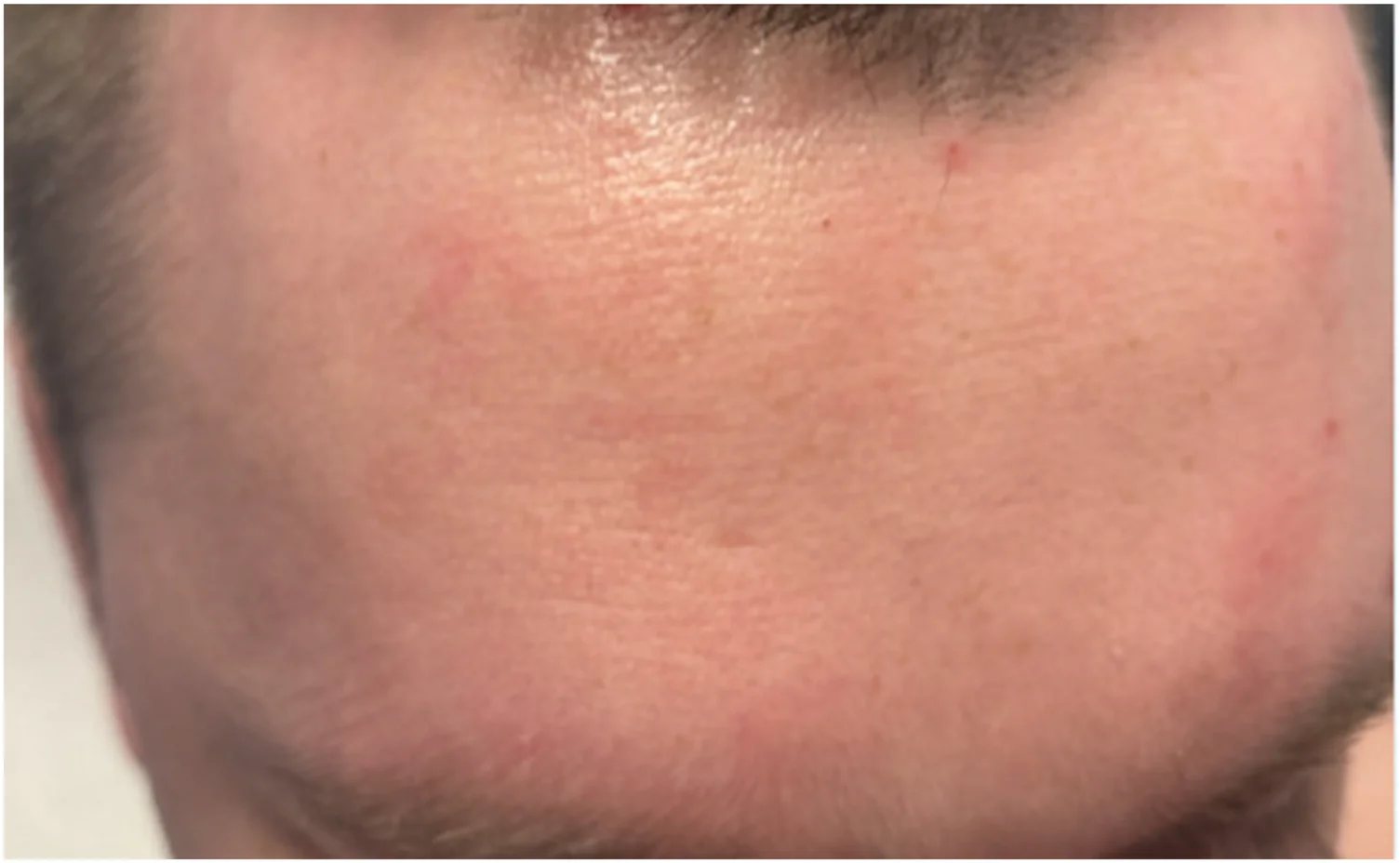
Facial involvement at 6 months follow-up showing mild residual eczema without excoriation or lichenification.

## Discussion

Management of moderate-to-severe atopic dermatitis can be challenging when conventional systemic therapies are contraindicated or poorly tolerated. This case highlights a clinically important scenario where therapeutic options are limited, and treatment sequencing decisions become critical.

Although tralokinumab and lebrikizumab are monoclonal antibodies that target the IL-13 pathway, they differ in their mechanisms of receptor interaction. Tralokinumab prevents IL-13 from binding to both the IL-13R*α*1 signalling receptor and the IL-13R*α*2 decoy receptor. Contrastingly, lebrikizumab has a higher binding affinity and selectively inhibits IL-13 signalling by preventing formation of the IL-4R*α*-IL-13R*α*1 receptor complex while preserving IL-13 interaction with the IL-13R*α*2 decoy receptor (8). This preserved interaction may facilitate receptor-mediated internalisation and degradation and may partly explain the differences in clinical responses between IL-13 inhibitors. However, the extent to which these mechanistic differences account for the differential response observed in this case remains hypothetical and cannot be established from a single case.

In this case, the patient showed minimal improvement with tralokinumab but experienced rapid and marked improvement following the initiation of lebrikizumab, with only mild recurrence of flare by six months. The initial reported improvement in pruritus following lebrikizumab is noteworthy, as itch is one of the most burdensome symptoms of atopic dermatitis and a major determinant of patient quality of life. However, baseline pruritus was not formally scored, and the reported improvement was not sustained, with NRS increasing from 3 at four months to 6 at six months. IL-13 has been implicated in neuroimmune pathways underlying pruritus in AD (9), with evidence suggesting that IL-13 signalling may sensitise peripheral sensory neurons to pruritogens and contribute to itch perception (10, 11). Whether differential modulation of this pathway meaningfully influences pruritus outcomes between IL-13 inhibitors warrants further exploration in larger studies and longer follow-up.

AD is increasingly recognised as a biologically heterogeneous disease with different immune pathways driving disease activity (12). This variation may influence individual responses to targeted therapies and may partly explain differing responses to the two IL-13 inhibitors observed in this case. Although intermittent therapies including topical corticosteroids and tacrolimus were used on an as-needed basis, the overall rapid improvement in disease severity following initiation of lebrikizumab remained relatively sustained by six months, supporting a true treatment effect rather than natural disease fluctuation.

While a delayed response to tralokinumab cannot be fully excluded, the lack of sustained clinical improvement, followed by rapid clinical response within four months of switching to lebrikizumab, supports a potential differential treatment effect. Although abnormal liver function tests limited treatment selection, they remained elevated despite modest improvement during treatment. There were no documented changes in alcohol intake that appeared sufficient to account for the marked clinical response observed following lebrikizumab. The absence of a washout period between therapies further supports that clinical improvement closely followed treatment initiation. Distinguishing primary from secondary treatment failure may have clinical relevance, as primary failure does not necessarily indicate class-wide inefficacy and may support consideration of intra-class switching.

Phase 3 clinical trials evaluating the efficacy of lebrikizumab in AD demonstrated rapid improvements in both skin clearance and pruritus, consistent with findings from earlier phase 2 studies (13–15). The rapid improvement in disease severity observed in our patient is consistent with trial data. Importantly, this case suggests that lack of response to one IL-13 inhibitor does not necessarily preclude response to another, although these findings are hypothesis generating and require confirmation in larger studies. This observation raises the possibility of intra-class heterogeneity among IL-13 inhibitors and may represent a reasonable approach for patients with limited therapeutic options.

This report has some limitations. As a single case, the findings are not generalisable. The absence of a washout period between biologic therapies may introduce uncertainty in attributing clinical response solely to lebrikizumab, although the prolonged lack of clinical response to tralokinumab and subsequent rapid improvement following treatment switch supports a true differential effect. Although topical corticosteroids and tacrolimus were used intermittently on an as-needed basis during lebrikizumab treatment, their contribution to the observed clinical response cannot be completely excluded. However, use was at a similar frequency during tralokinumab treatment, when significant flares occurred, making topical therapy less likely to be the primary driver of improvement. Lastly, post-treatment photographs of identical baseline anatomical sites were unavailable, limiting direct visual comparison of clinical improvement. Nevertheless, the observed clinical response was supported by objective measures, including EASI, POEM, and DLQI.

## Conclusion

As the therapeutic landscape for AD continues to expand, clinicians are increasingly faced with decisions regarding treatment sequencing when intolerance, contraindications, or suboptimal responses occur. This case suggests that primary failure to tralokinumab does not preclude a meaningful clinical response to lebrikizumab and raises the possibility of heterogeneity in clinical response among IL-13 inhibition. Sequential IL-13 inhibition may be considered in patients with limited systemic options. Further research, including larger studies, is needed to determine whether clinical or molecular factors may help predict response to individual IL-13 inhibitors and guide more personalised treatment strategies in AD.

## Data Availability

The original contributions presented in the study are included in the article/Supplementary Material, further inquiries can be directed to the corresponding author.
